# Transcriptional repression of the human collagenase-1 (MMP-1) gene in MDA231 breast cancer cells by all-*trans*-retinoic acid requires distal regions of the promoter

**DOI:** 10.1038/sj.bjc.6690037

**Published:** 1999-01

**Authors:** U Benbow, J L Rutter, C H Lowrey, C E Brinckerhoff

**Affiliations:** Department of Medicine, Dartmouth Medical School, Hanover, NH 03755, USA; Department of Pharmacology and Toxicology, Dartmouth Medical School, Hanover, NH 03755, USA; Department of Biochemistry, Dartmouth Medical School, Hanover, NH 03755, USA

**Keywords:** DNAase I hypersensitivity, transfection, retinoic acid receptors/retinoid X receptors, gelshifts

## Abstract

In the present study, we investigated the mechanisms controlling constitutive transcription of collagenase-1 and its repression by all-*trans*-retinoic acid (RA) in the highly invasive metastatic and oestrogen-receptor-negative breast cancer cell line MDA231. A combination of in vivo and in vitro experiments that include DNAase I hypersensitivity assays, transient transfection of collagenase-1 promoter constructs, and electrophoretic mobility shift assays implicate several PEA3 sites, binding sites for Ets-related transcription factors, in the constitutive expression of the human collagenase-1 promoter. Transient transfection of promoter constructs linked to the luciferase reporter, along with gel retardation assays, revealed that repression of collagenase-1 transcription by RA is not dependent on the proximal AP-1 site, but, rather, requires sequences located in distal regions of the promoter. Transcriptional analyses and electrophoretic mobility shift assays suggest that the PEA3 site located at –3108 bp facilitates, at least in part, the transcriptional repression of the human collagenase-1 gene in MDA231 cells. We conclude that collagenase-1 repression in MDA231 cells occurs by a novel regulatory pathway that does not depend on the proximal AP-1 site at –73 bp, but does depend on distal regions in the collagenase-1 promoter. © 1999 Cancer Research Campaign


*British Journal of Cancer* (1999) **79**(2), 221–228
© 1999 Cancer Research Campaign

### Transcriptional repression of the human collagenase-1 (MMP-1) gene in MDA231 breast cancer cells by all-*trans*-retinoic acid requires distal regions of the promoter

**U Benbow[1], JL Rutter[2], CH Lowrey[1,2] and CE Brinckerhoff[1,3]**

[1]Department of Medicine, Dartmouth Medical School, Hanover NH 03755, USA; [2]Department of Pharmacology and Toxicology, Dartmouth Medical School, Hanover NH 03755, USA; [3]Department of Biochemistry, Dartmouth Medical School, Hanover NH 03755, USA

**Summary** In the present study, we investigated the mechanisms controlling constitutive transcription of collagenase-1 and its repression by all-*trans*-retinoic acid (RA) in the highly invasive metastatic and oestrogen-receptor-negative breast cancer cell line MDA231. A combination of in vivo and in vitro experiments that include DNAase I hypersensitivity assays, transient transfection of collagenase-1 promoter constructs, and electrophoretic mobility shift assays implicate several PEA3 sites, binding sites for Ets-related transcription factors, in the constitutive expression of the human collagenase-1 promoter. Transient transfection of promoter constructs linked to the luciferase reporter, along with gel retardation assays, revealed that repression of collagenase-1 transcription by RA is not dependent on the proximal AP-1 site, but, rather, requires sequences located in distal regions of the promoter. Transcriptional analyses and electrophoretic mobility shift assays suggest that the PEA3 site located at –3108 bp facilitates, at least in part, the transcriptional repression of the human collagenase-1 gene in MDA231 cells. We conclude that collagenase-1 repression in MDA231 cells occurs by a novel regulatory pathway that does not depend on the proximal AP-1 site at –73 bp, but does depend on distal regions in the collagenase-1 promoter.

**Keywords:** DNAase I hypersensitivity; transfection; retinoic acid receptors/retinoid X receptors; gelshifts

Matrix metalloproteinases (MMPs) have been implicated in multiple physiological and pathological processes related to extracellular matrix turnover, such as normal growth and development, wound healing, angiogenesis, joint destruction in arthritis, and tumour invasion and metastasis (Woessner, 1991; Stetler-Stevenson et al, 1993; Birkedal-Hansen et al, 1995; Crawford and Matrisian, 1996; Nagase, 1996). In the MMP family, the gelatinases [gelatinase A (MMP-2) and gelatinase B (MMP-9)] are thought to play major roles in the invasive and metastatic behaviour of cancer cells because of their ability to cleave the helical domain of type IV collagen, a principal structural component of the basement membrane (Stetler-Stevenson et al, 1993; Dickson et al, 1994). However, invasion also requires that tumour cells traverse the extracellular matrix, which is composed primarily of collagens type I and III. The collagenases [interstitial collagenase (collagenase-1, MMP-1), neutrophil collagenase (MMP-8), collagenase-3 (MMP-13) and MT1-MMP] are the only members of the metalloproteinase family able to digest collagen type I at neutral pH (Jeffrey, 1986; Ohuchi et al, 1997). Furthermore, bone, which is composed principally of collagens type I and III, is one of the most common sites of metastasis in breast cancer (Sugarbaker et al, 1981). Thus, the processes of tumour invasion and metastasis include the degradation of collagen by collagenase-1.

Stromal cell production of MMPs has been implicated as the major contributor to overall tumour metalloproteinase activity (Crawford and Matrisian, 1994; MacDougal and Matrisian, 1995). Although collagenase-1 has been associated with stromal cells in some breast cancers (Heppner et al, 1996; Dickson et al, 1994), this enzyme is also constitutively secreted from several tumour cells, including highly invasive and metastatic breast cancer cell lines, such as the oestrogen-receptor-negative MDA231 cells (Templeton et al, 1990). This finding indicates that the tumour cells, themselves, can contribute to their own invasiveness, and suggests that decreasing collagenase-1 production by these cells may decrease their invasive potential.

The vitamin A analogues, all-*trans*-retinoic acid and the synthetic retinoids, inhibit transcription of the collagenase gene by binding to nuclear receptors, the retinoic acid receptors (RARs) and retinoid X receptors (RXRs) (Mangelsdorf et al, 1994, 1995; Schroen and Brinckerhoff, 1996*a*), each with subtypes classified as α, β and γ. The RARs/RXRs display specific patterns of expression throughout development, and contribute to the process of cell growth and differentiation (Mangelsdorf et al, 1994; Schroen and Brinckerhoff, 1996*a*). Additionally, they prevent chemically induced carcinogenesis in experimental animals (Moon et al, 1992; Anzano et al, 1994) and inhibit proliferation of a variety of normal and neoplastic cell types in vitro (Lotan et al, 1980). More recently, the effectiveness of retinoids in the treatment and prevention of a number of human cancers, including breast cancer, has been established (Castaigne et al, 1990; Costa, 1993; Pastorini et al, 1993; Hong and Lippman, 1995; Lippman et al, 1995).

Previously, we demonstrated that the inhibition of collagenase-1 synthesis in fibroblasts treated with RA occurs at the transcriptional level (reviewed in Vincenti et al, 1996; Schroen and Brinckerhoff, 1996*a*; Benbow and Brinckerhoff, 1997). This inhibition is mediated

*Received 2 March 1998*
*Revised 16 June 1998*
*Accepted 13 July 1998*

*Correspondence to:* CE Brinckerhoff, Department of Biochemistry, Dartmouth Medical School, Hanover NH 03755, USA

through a variety of mechanisms that involve the activator protein 1 (AP-1) site at ~ –70 bp (Lafyatis et al, 1990; Schule et al, 1991; Pan et al, 1992, 1995; Schuchard et al, 1993; Schroen and Brinckerhoff, 1996*b*; Caelles et al, 1997). To determine whether retinoids exert similar effects in breast cancer cells, we investigated the role of all-*trans*-retinoic acid (RA) on the repression of collagenase-1 in the oestrogen-receptor-negative MDA231 breast carcinoma cells, a cell line that is resistant to the growth inhibitory effects of retinoid treatment (Sheikh et al, 1994).

We report that (1) constitutive expression of collagenase-1 in MDA231 cells correlates with several PEA3 sites in the distal promoter, (2) repression of collagenase-1 by RA does not depend on the proximal AP-1 site and (3) repression by RA is mediated, at least in part, by a PEA3 site located at –3.108 bp. Our data suggest that, compared with fibroblasts, RA-mediated repression of the collagenase-1 gene in MDA231 cells occurs by a novel mechanism.

### MATERIALS AND METHODS

### Cell culture and Western analysis

The human breast cancer cell line MDA231 was provided by Dr Lance Liotta at the NIH, Bethesda, MD, USA. Cell cultures were propagated in Dulbecco's modified Eagle medium (DMEM; Gibco), supplemented with 10% fetal calf serum (FCS; Gibco), penicillin (100 U $ml^{-1}$) and streptomycin (100 µg $ml^{-1}$). At confluency, cells were washed with Hanks' balanced salt solution (HBSS; Gibco) and incubated with or without all-*trans*-retinoic acid (RA; Sigma; $10^{-6}$ M) in the presence of DMEM and lactalbumin hydrosylate (LH; 0.20%). This concentration of RA inhibits collagenase-1 synthesis in fibroblasts and human tumour cells (Pan et al, 1992; Agadir et al, 1997). Culture medium (1 ml) was precipitated with 0.5 ml of 10% trichloroacetic acid (TCA) for 30 min on ice. Proteins were pelleted and resuspended in sodium dodecyl sulphate polyacrylamide gel electrophoresis (SDS-PAGE) sample buffer, electrophoresed on 7.5% SDS-PAGE minigels, transferred to Immobilon-P membranes (Millipore, Bedford, MA, USA) using a Trans-Blot Cell (BioRad). Collagenase protein was detected as previously described (Rutter et al, 1997). Briefly, the membranes were blocked for 1 h with 10% FCS, then probed overnight with anti-(human) collagenase antibody at a dilution of 1:10.000. The membranes were then washed and specific antibody binding was detected using the Vectastain ABC kit (Vector Laboratories, Burlingame, CA, USA).

### Northern analysis

Total cellular RNA was isolated using Trizol LS Reagent (Gibco). Briefly, cells were scraped from the plates, pelleted and lysed in Trizol LS Reagent. After chloroform extraction, the RNA was recovered by precipitation with isopropanol. Total RNA was quantitated by optical density, and 10 µg of RNA was subjected to Northern analysis as previously described (Sambrook et al, 1989). Northern blots were hybridized with an [α-$^{32}$P]dCTP-labelled probe for 20 h at 56°C (0.2 × SSC, 0.5% SDS). Blots were washed twice at room temperature with 2 × SSC, followed by two 30-min washes at 56°C (0.2 × SSC, 0.5% SDS) and autoradiographed. The human collagenase probe and probes for RARs, RXR-α and AP-1 family members have been described previously (Pan et al, 1992; White and Brinckerhoff, 1995). Glyceraldehyde-3-phosphate dehydrogenase (GAPDH) was used as a loading control.

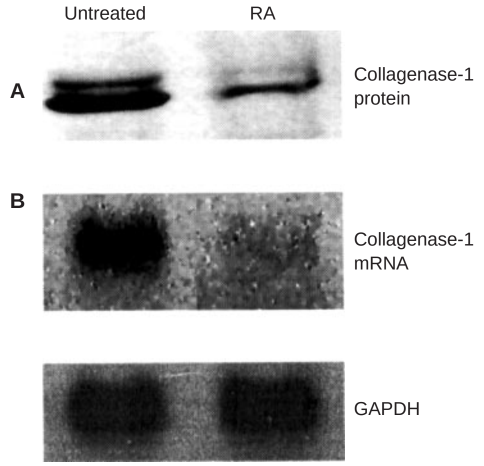

**Figure 1** Suppression of collagenase-1 by RA in MDA231 cells. MDA231 cells in DMEM/LH were treated with all-*trans*-RA ($10^{-6}$ M) for 18 h. (**A**) Culture medium (1 ml) was subjected to TCA precipitation and Western analysis with sheep antiserum specific to human collagenase-1. (**B**) Total RNA (10 µg) was subjected to Northern analysis. The membrane was hybridized with a probe specific for human collagenase-1, and with GAPDH as loading control

### Transfection and luciferase assays

The reporter plasmids cloned into the PXP2 luciferase vector have been described previously (Rutter et al, 1997). Plasmid DNA was prepared using the Qiagen maxiprep kit (Qiagen). The plasmids were transiently transfected using Lipofectamine (Gibco). Briefly, cells were plated at $1.75 \times 10^5$ in six-well cluster plates in DMEM containing 10% FCS. The following day, the cells were transfected with 5 µg DNA and 5 µl Lipofectamine per plate. After 5 h, 1 ml of DMEM containing 20% FCS was added. After 18 h, the cells were washed three times with HBSS and incubated in serum-free DMEM/LH medium alone or in the presence of $1 \times 10^{-6}$ M RA. Cell lysates were harvested 24 h after treatment and luciferase activity was determined using a temperature controlled luminometer as described previously (Rutter et al, 1997). The values were normalized to the protein content of the harvested lysate using a modification of the Lowery Assay (DcProtein Assay, BioRad, Melville, NY, USA). Each transfection was carried out in triplicate; each plasmid was transfected in at least four separate experiments using different DNA preparations. Transfection efficiency was determined by Hirt's analysis (Ausuble et al, 1987; Sambrook et al, 1993; White and Brinckerhoff, 1995).

### DNAase I hypersensitivity assays

DNAase I hypersensitivity assays were performed on nuclei isolated from untreated cells as described previously (Lowrey et al, 1992). Nuclei (200 µg DNA per reaction) were digested in a total volume of 200 µl with DNAase I at 0, 0.1, 0.25, 0.5, 1.0, 1.5 and 2.0 µg $ml^{-1}$. Reactions were performed at 37°C for 10 min. For Southern blotting, genomic DNA was digested with *Hin*dIII and *Xba*I. This digestion releases a 4.6-kb genomic fragment which has the same 5′ end as the full-length promoter construct. A 120-bp *Eco*RI/*Xba*I fragment corresponding to the N-terminal region of the collagenase cDNA (Brinckerhoff et al, 1987) was used as a probe for Southern blot assays.

 © Cancer Research Campaign 1999

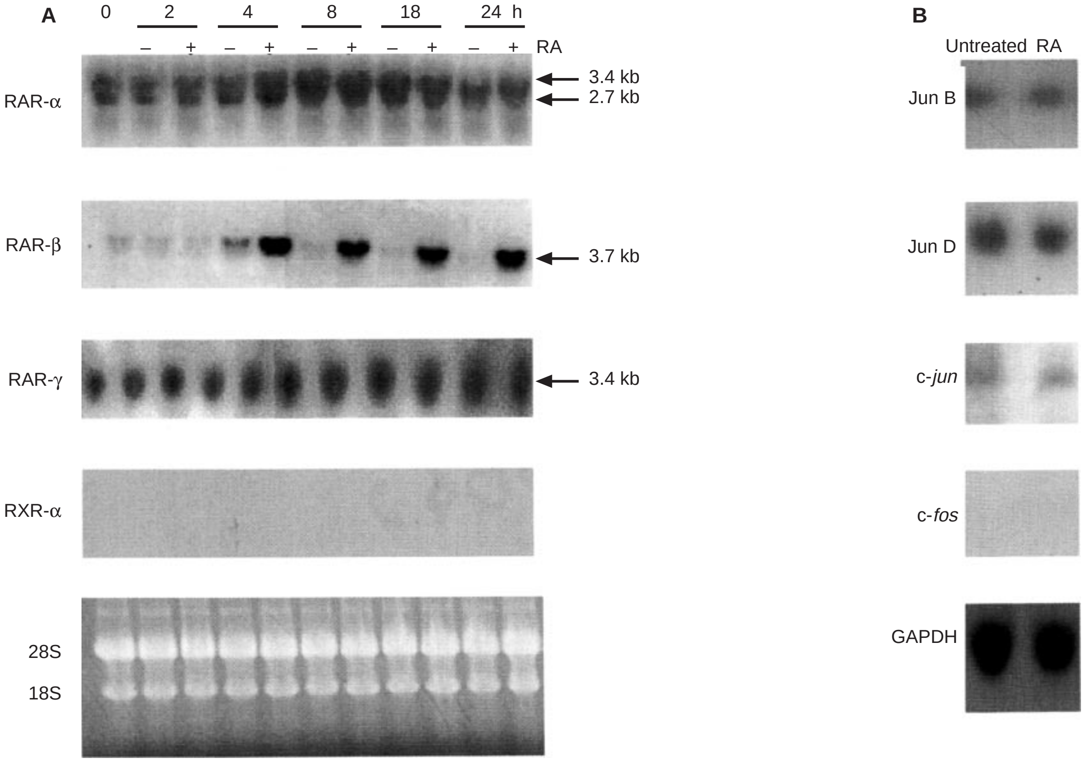

**Figure 2** Expression of RARs/RXR and AP-1 transcription factors in MDA231 cells. Confluent cultures of MDA231 cells in serum-free DMEM/LH medium, were left untreated or were treated with RA ($10^{-6}$ M). At the time points indicated, total RNA was harvested and 20 µg were subjected to Northern analysis. (**A**) Time course for the expression of RAR and RXR mRNAs in MDA231 cells. (**B**) RNA analysis (18 h time points) of members of the *fos* and *jun* family of transcription factors. Control for loading is shown by ethidium bromide staining of 18S and 28S RNAs or by hybridization with a cDNA for GAPDH

### Electrophoretic mobility shift assays (EMSAs)

Oligonucleotides corresponding to human collagenase 5′ flanking DNA were used as probes in EMSA assays: PEA3 from –2989/–3024 (top 5′-TGAATGGCCATATGTAGGAA GAATACACAC-CGTGAG-3′, bottom 5′-ATTCTTCCTACATATGGCCATTCA-3′; AP-1 site from –83/–57 (top 5′-AAAGCATGAGTCACACAGCC-CTCCAGCT-3′, bottom 5′AGCTGG AGGGCTGTGTGACTCAT-GCTTT-3′); PEA3 from –3818/–3852 (top 5′-TTGGGAGTC-TGAGGCAGGAACATTGCTTAAGCCCA-3′, bottom 5′-TGGG-CTTAAGCAATGTTCCTGCCTCAGACTCCCAA-3′); nonspecific oligonucleotide [top, 5′ AGCTTGCTCAGGCTAT-3′ and bottom 5′ ATAGCCTGAGCAAGCT-3′ (Pan et al, 1992)]. Complementary strands were annealed and end labelled with (γ-$^{32}$P)ATP using T4 kinase. Labelled oligonucleotides (30. 000 c.p.m.) were incubated on ice for 30 min with nuclear extracts (5 µg) of untreated or RA-treated cells in 10 µl binding reaction as described previously (Schroen and Brinckerhoff, 1996*b*). Nuclear extracts were harvested after 18 h RA treatment. The 18-h time point was determined by time course analysis. Samples were electrophoresed at 150 V on 5% acrylamide gels under non-reducing conditions, dried and autoradiographed at –70°C using DuPont autoradiographic film.

### RESULTS

### All-*trans*-retinoic acid (RA) suppresses collagenase-1 gene expression in the MDA231 breast cancer cell line

MDA231, a non-oestrogen-dependent breast carcinoma cell line, expresses collagenase-1 constitutively (Templeton et al, 1990). As a first step in investigating the mechanism(s) controlling collagenase-1 expression in these cells, we confirmed these previous findings. RNA and media were harvested from confluent cells that were cultured in serum-free conditions for 24 h, with or without RA ($1 \times 10^{-6}$ M). Figure 1 demonstrates that in these MDA231 cells (1) collagenase-1 mRNA is produced constitutively, (2) RA represses both collagenase-1 mRNA and protein and (3) levels of collagenase protein correlate with levels of collagenase-1 mRNA (Vincenti et al, 1996). Densitometric quantification of mRNA and protein revealed a 55% and 51% decrease respectively (data not shown). In addition, in agreement with Sheikh et al (1994), RA at a concentration of $1 \times 10^{-6}$ M was not cytotoxic and had no effect on cell proliferation (data not shown).

### All-*trans*-retinoic acid induces expression of RAR-β, but does not affect the expression of other RARs/RXRs or of AP-1 binding proteins in MDA231 cells

It has previously been demonstrated that RA is a negative regulator for AP-1 responsive genes (Nicholson et al, 1990; Schule et al, 1991; Yang-Yen et al, 1991; Pan et al, 1992; Schroen and Brinckerhoff, 1996*a*). Several mechanisms may account for the interference of AP-1 activity by RA. First, RA decreases FOS and JUN mRNA levels in rabbit fibroblasts (Lafyatis et al, 1990, Pan et al, 1992), whereas increasing mRNA levels for RARs and RXRs (Pan et al, 1995). Second, RAR/RXRs may bind directly to FOS/JUN and sequester the AP-1 proteins (Nicholson et al, 1990; Schule et al, 1991; Salbert et al, 1993); third, nuclear hormone receptors antagonize AP-1 by inhibition of the Jun amino-terminal kinase (JNK) pathway (Caelles et al, 1997).

*© Cancer Research Campaign 1999*

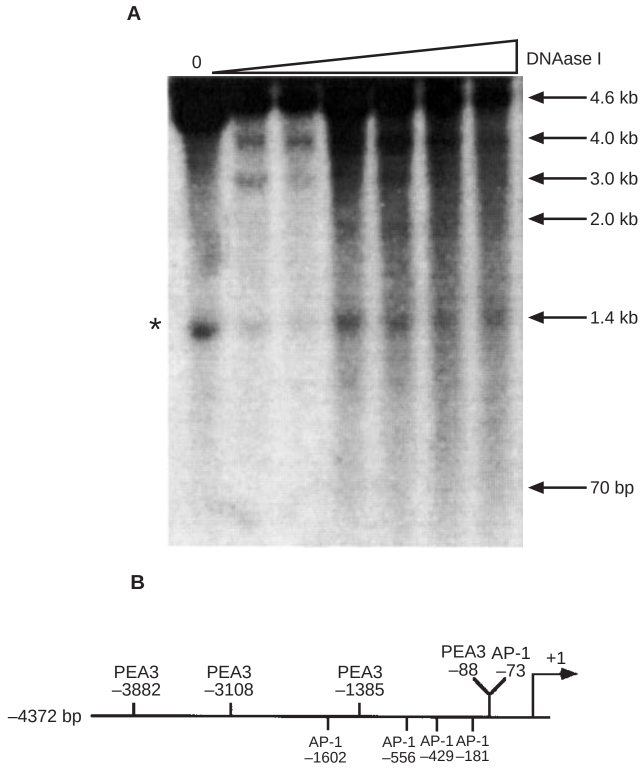

**Figure 3** Identification of hypersensitivity sites in the human collagenase-1 promoter. Increasing amounts of DNAase I was added from left to right. (**A**) DNAase I hypersensitivity sites in the human collagenase-1 promoter associated with basal transcription in MDA231 cells are indicated by arrows. The band marked with an asterisk (*) may not denote a true hypersensitive site because this band is also present in the absence of DNAase I (lane 1). (**B**) Location of the various AP-1 and PEA3 sites throughout the collagenase-1 promoter

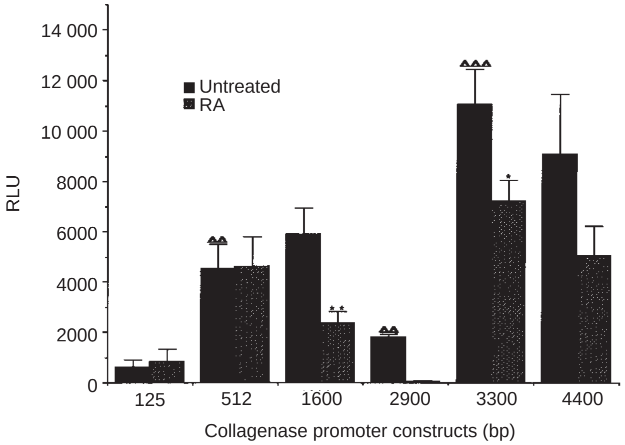

**Figure 4** Constitutive transcription of the human collagenase-1 promoter and RA repression of promoter activity in MDA231 cells. MDA231 cells were transiently transected with 5 μg of the indicated promoter constructs together with 5 μl LipofectAmine, added to each well of $1.75 \times 10^5$ cells in DMEM/LH. Cells were harvested after 18 h and luciferase activity was read on an ML2250 Microtiter Plate Luminometer (Dynatech Laboratories). Activity is expressed as relative light units (RLUs). Each transfection was carried out in quadruplicate. Error bars represent the standard deviation of the mean. The increase in constitutive expression seen with the 512-bp vs the 125-bp construct was very significant ($\Delta\Delta P < 0.0054$) or extremely significant ($\Delta\Delta\Delta P < 0.0003$) when compared with the 3300-bp construct. RA treatment resulted in a very significant repression of collagenase-1 transcription ($^{**}P < 0.0061$ for 1600-bp promoter construct) and a significant repression ($^{*}P < 0.014$) for the 3300-bp construct when compared with the 125-bp construct

Therefore, to investigate the possible role of RARs and RXRs in collagenase-1 repression in MDA231 cells, we performed Northern analyses to determine the effects of RA on expression of these receptors (Figure 2A). We found that mRNAs for RAR-α and RAR-γ were constitutively expressed and were not regulated by RA. RXR-α was not detectable and was not induced by RA. In contrast, RA increased the steady-state mRNA level for RAR-β within 4 h, and high levels were maintained over a 24-h period. It has been reported that, compared with fibroblasts, the expression of RAR-β is absent or down-regulated in most breast cancer cell lines (Seewaldt et al, 1995; Pan et al, 1995). We note, however, that although constitutive levels of RAR-β appear to be lower than levels of RAR-α or RAR-γ in untreated cells, it was induced by RA, suggesting that RAR-β may play a role in suppression of collagenase-1 transcription in MDA231 cells. In contrast, the inability of RA to regulate RAR-α supports the studies by Sheikh et al (1994) in which dysregulation of RAR-α in these cells was responsible for the failure of RA to inhibit cell growth.

Because the inhibition of collagenase-1 and of stromelysin-1 by RA in fibroblasts occurs primarily, but not exclusively, through the AP-1 site at ~ –70 bp, we examined the effect of RA treatment on levels of mRNA for members of the AP-1 family of transcription factors. c-*Jun*, junB and junD were present constitutively in untreated cells, whereas c-*fos* was undetectable, and treatment of cells with RA for 18 h did not change the level of mRNA for these AP-1 factors (Figure 2B). These results indicate that repression of collagenase-1 in MDA231 cells is not mediated by down-regulation of AP-1 binding factors.

### Identification of DNAase I hypersensitivity sites in the collagenase-1 promoter

To determine the regulatory regions in the collagenase-1 promoter that are involved in the constitutive transcription of collagenase-1 in MDA231 cells, we performed DNAase I hypersensitivity assays. This in vivo assay is able to detect and localize domains of altered chromatin structure such as those formed by the binding of transcription factors to enhancers or promoters, and allows the screening of large regions of genomic DNA for potential regulatory elements (Elder et al, 1990; Stamatoyannopolus et al, 1995). The pattern of DNAase I digestion of nuclei from untreated cells is shown in Figure 3A. This assay detected the expected parental fragment of 4600 bp and four major sub-bands at approximately –4000 bp, –3000 bp, –2000 bp and –1400 bp. The band at –1400 bp is also present in the DNAase untreated lane and may, therefore, not constitute a true hypersensitive site (Lowrey et al, 1992), even though transcriptional studies suggest otherwise (see below). Alignment of the regions identified by DNAase I treatment with the 4372-bp human collagenase-1 promoter sequence reveals the presence of several PEA3 sites (5′-C/AGGAA/TG-3′) (Figure 3B), which bind members of the Ets family of transcription factors (Gutman and Wasylyk, 1990). Because PEA3 sites at –3108 bp and –3882 bp are localized in a region of the promoter that is devoid of other known *cis*-acting elements (Rutter et al, 1997), it is possible that these sites contribute to constitutive transcription of the collagenase-1 promoter.

*© Cancer Research Campaign 1999*

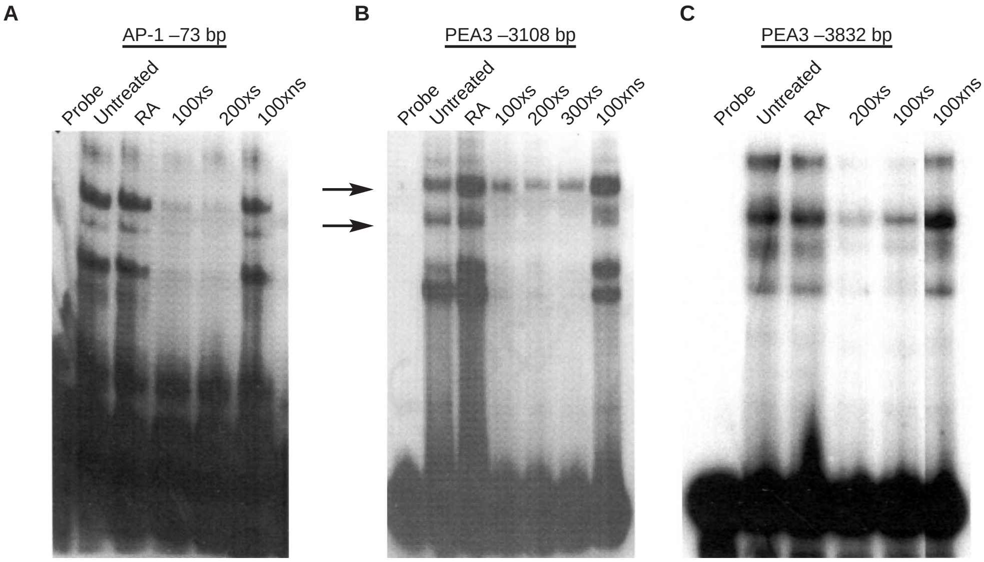

**Figure 5** Specific complex formation between nuclear proteins and radiolabelled fragments of the human collagenase-1 promoter. Gel shift analysis of nuclear proteins and radiolabelled probes containing the (**A**) AP-1 site at –73 bp, (**B**) PEA3 site at –3108 bp, and (**C**) PEA3 site at –3832 bp. Complexes were formed using 5 µg of nuclear extracts from untreated and RA treated cells with radiolabelled probes as indicated. The arrows in (**B**) point out a change in the binding pattern in RA-treated extracts; s, specific oligonucleotide; ns, non-specific oligonucleotide; probe, free probe

### Upstream sequences enhance both constitutive transcription and RA-mediated repression of the collagenase-1 promoter

To further define regions of the collagenase-1 promoter that participate in constitutive transcription, we used transient transfection of a series of collagenase-1 promoter fragments linked to the luciferase reporter gene. The smallest construct (125 bp), containing an AP-1 site at –73 bp and a PEA3 site at –88 bp, displays only minimal activity (Figure 4). A 6.5-fold increase in activity ($\Delta\Delta P < 0.0054$) was observed between 125 bp and 512 bp. This increase may be due to the presence of two putative AP-1 sites, at –181 bp and –429 bp of the promoter (Figure 3B; Rutter et al, 1997). Thus, sequences in the proximal promoter, other than the AP-1 site at –73 bp, have a role in constitutive transcription. However, transcription decreased approximately threefold between 1600 bp and 2900 bp ($\Delta\Delta P < 0.0027$), a region of the promoter containing several potential repressor elements (Rutter et al, 1997). Fragments of 3300 bp or larger showed a sixteenfold increase in activity over the 125 bp construct, thereby overcoming the repression observed with the 2900 bp construct. These studies, together with the DNAase I hypersensitivity data, support the hypothesis that upstream elements, i.e. PEA3 sites located between –3000 bp and –4000 bp, play a role in transcriptional activity of the human collagenase-1 promoter.

We then examined the ability of RA to repress transcription. Treatment of the cells with RA resulted in significant repression of the collagenase-1 promoter only with fragments larger than 512 bp (*$P < 0.038$) (Figure 3), suggesting that the inhibitory effects of RA are independent of the proximal AP-1 site. With larger fragments containing 1600 bp, and 3300 bp of the promoter, a significant repression of 1.8- and 1.5-fold (**$P < 0.0061$ and *$P < 0.014$ respectively) by RA was also observed. Compared with the 1600 bp construct, 2900 bp of the collagenase-1 promoter shows a sharp decrease in constitutive expression, and treatment with RA resulted in an additional inhibition of promoter activity of approximately eightfold. Because this region contains several silencing elements, it is possible that their function may be enhanced in the presence of RA. However, most importantly, this constitutive repression is overcome with larger promoter fragments, indicating the presence of positive regulatory elements that override the negative ones (Figure 4). The fold of RA repression observed with these larger constructs, including the 4400-bp fragments was similar to that seen with the 1600-bp construct, suggesting that the RA responsive element(s) is located upstream of 512 bp in distal regions of the promoter.

### The PEA3 site at –3108 bp, but not the AP-1 site, binds nuclear proteins induced by all-*trans*-retinoic acid

The inability of RA to suppress AP-1 proteins was further investigated by gel mobility shift analyses (EMSA). We compared the pattern of nuclear proteins from untreated and RA-treated MDA231 cells that bound to an oligonucleotide containing the AP-1 site at –73 bp. In contrast to previous findings in fibroblasts (Pan et al, 1995), we found that RA treatment of this breast cancer cell line did not alter the pattern of AP-1 binding proteins (Figure 5A). The inability of RA to alter the complex of nuclear proteins binding to the AP-1 site supports the hypothesis that this site does not participate in repression of collagenase-1 activity in the MDA231 cells. This hypothesis is further strengthened by the data presented in Figures 3 and 4B, which illustrate that transcription of a small promoter fragment is not suppressed by RA, and that the constitutive expression of c-*jun*, junB and JunD in the MDA231 cells is not altered by RA.

Because our DNAase I hypersensitivity assays and the transcriptional analysis implicated regions in the distal promoter that contain PEA3 sites in constitutive transcription, we questioned whether these sites may be involved in repression by RA. We used EMSA to examine the role of the individual PEA3 sites in the binding of nuclear extracts from both untreated MDA231 cells or from cells treated with RA. We found specific binding to

© Cancer Research Campaign 1999 

oligonucleotides containing a PEA3 site at –88 bp, –1381 bp (data not shown) and –3832 bp (Figure 5B) by nuclear extracts from untreated and RA-treated cells. Importantly, however, the binding pattern did not change when cells were treated with RA. In contrast, an oligonucleotide encompassing the PEA3 site at –3108 bp showed an increase in binding activity with extracts from RA-treated cells. This RA-induced binding could be completed by self, but not by a non-specific oligonucleotide (Figure 5C). These findings suggest that the PEA3 site at –3108 bp may, at least in part, mediate RA repression of collagenase-1 in MDA231 cells. Similarly, Schneikert et al (1996) reported that inhibition of collagenase-1 and stromelysin-1 via the androgen receptor is mediated by members of the Ets family of transcription factors (see Discussion section).

### DISCUSSION

In this study, we investigated the mechanisms controlling constitutive transcription of collagenase-1 and its repression by all-*trans*-retinoic acid (RA) in the highly invasive and metastatic breast cancer cell line MDA231 (Templeton et al, 1990; Bae et al, 1993). Low constitutive expression of the collagenase-1 promoter was observed with a small (125 bp) promoter fragment, whereas a fragment encompassing 512 bp of the promoter showed a significant increase in transcriptional activity. This increase may be due to several AP-1 sites present in the region between –125 bp and –512 bp (Figure 3B) (Rutter et al, 1997). Possibly, these sites cooperate with the proximal AP-1/PEA3 elements (Benbow and Brinckerhoff, 1997), resulting in an increased transcriptional activation. This transcription may be further enhanced by elements located in the distal promoter, including PEA3 sites at –1381 bp, –3108 bp and –3832 bp, whose location correlates with the pattern of DNAase I hypersensitive sites (Figure 3). We also note that constitutive transcription of the collagenase-1 promoter was significantly reduced ($\Delta\Delta P < 0.0027$) between –1600 and –2900 bp. This region contains several putative silencer elements (Rutter et al, 1997), which may account for the reduction in activity. Importantly, however, positive regulatory elements, which reside 5′ of –2900 bp can overcome this repression, indicating that the expression of the endogenous collagenase-1 gene is the net total of positive and negative regulators.

Along with the DNAase I hypersensitivity studies, transient transfection and gel mobility analyses also implicated a role for a PEA3 site in the regulation of collagenase-1 in MDA231 cells. Proteins bound constitutively to these sites, and binding was specific as shown by competition with a self but not with a non-specific oligonucleotide. Interestingly, expression of the PEA3 group of Ets-related transcription factors in human breast cancer cells has been observed (Baert et al, 1997; Benz et al, 1997). These genes were expressed at high levels in oestrogen-receptor-negative cells, including MDA231, when compared with oestrogen-receptor-positive cell lines. These Ets-related transcription factors are thought to have a potential role in the regulation of growth and the progression of breast cancer cells, although the target genes for these transcription factors are not known (Baert et al, 1997). Our data suggest that the collagenase-1 promoter is a primary target.

The importance of upstream regions in the regulation of the human collagenase-1 promoter has been shown previously. Activation of the collagenase-1 gene by interleukin 1β (IL-1β) in both fibroblasts and BC8701 breast cancer cells is regulated by regions in the distal promoter (Rutter et al, 1997). In addition, Doyle et al (1997) reported that the transcriptional induction of collagenase-1 by PMA in differentiated monocyte-like (U937) cells is regulated not only by the proximal AP-1 site but also by a distal CCAAT/enhancer-binding protein-β site (C/EBP-β) located between –2010 bp and –1954 bp. Thus, it is becoming increasingly evident that in addition to the proximal AP-1/PEA3 elements, transcriptional regulation of the collagenase-1 gene depends on distal regions of the promoter (Benbow and Brinckerhoff, 1997).

Treatment of MDA231 cells with RA decreased collagenase-1 mRNA and protein (Figure 1), but did not change the levels of AP-1 transcription factors as shown by Northern analyses and by EMSA (Figures 2B and 5A). This observation is in agreement with work from van der Burg et al (1995), which showed an overall increase in AP-1 protein activity in hormone-independent human breast carcinoma cell lines and an inability of RA to repress AP-1 transactivation in the presence of RA. This may be due to the inhibition of the JNK cascade by RARs/RXRs, normally involved in the activation of AP-1 proteins (Caelles et al, 1997). Further evidence of AP-1-independent repression by RA in the MDA231 cells comes from the transfection data (Figure 4), which indicate that expression of a minimal promoter (125 bp) is not altered by RA, and from the observation that the binding of nuclear proteins to an oligonucleotide containing the proximal AP-1 was not affected by RA treatment (Figure 5A). However, RA repression does correlate with regions located in more distal regions of the promoter, including an element(s) between –512 bp and –1600 bp and the PEA3 element at –3108 bp (Figures 4 and 5B). Although the region between –3462 bp and –4054 bp of the collagenase promoter contains a number of putative silencer elements, the region between –2972 bp and –3462 bp contains only a cyclic AMP responsive element-binding protein (CREB) binding site, in addition to the PEA3 element at –3108 bp (Rutter et al, 1997). It is possible, therefore, that the presence of these silencers allows constitutive binding of proteins to the PEA3 site at –3832 bp, but does not allow the interaction of additional regulatory proteins due, perhaps, to steric hindrance.

In contrast to our findings, Agadir et al (1997) reported that retinyl methyl ether (RME) and RA down-regulate transcription of AP-1 activity induced by phorbol esters and growth factors in human breast cancer cells. The discrepancy may be explained by the fact that our studies were conducted in the context of a full-length collagenase-1 promoter, rather than with an isolated AP-1 site. Furthermore, Agadir et al (1997) induced AP-1 activity by treatment with phorbol esters, and treatment with RA may have sequestered this activity (Schroen and Brinckerhoff, 1996*b*). Although we did not see a change in AP-1 binding activity after RA treatment, we did observe that RA treatment increased the binding activity to an oligonucleotide spanning the PEA3 site at –3108 bp. Studies from Wasylyk et al (1992) pointed out that nucleotides adjacent to the core PEA3 site (GGA) can influence which proteins can bind to this site and, thus, it is likely that these adjacent sequences permit differential binding of proteins to the PEA3 site at –3108 bp vs. the site at –3832 bp. Nonetheless, we were not able to identify the proteins bound to the –3108 bp site, perhaps because of the limitations of antibodies available. However, in support of our findings, Schneikert et al (1996) reported that androgen negatively regulates the expression of several MMPs, including collagenase-1, in prostate cancer cells. Sequences in the collagenase-1 gene that mediate this effect did not encompass the regulatory AP-1 site at –73 bp but, rather, depended on a motif that binds ERM, a member of the Ets family

© Cancer Research Campaign 1999

of transcription factors. Thus, their findings set a precedent for non-AP-1-mediated repression of MMP gene expression by RA and our data support this concept.

High levels of expression of the Ets-related proteins, ERM and ER81, are present in MDA231 and other oestrogen-receptor-negative aggressive cell lines. They are not, however, present in oestrogen-receptor-positive cell lines such as MCF-7 cells (Baert et al, 1997), suggesting that this family of proteins has a role in the regulation of tumour progression in breast cancer. Indeed, Higashimo et al (1995) reported that the Ets-related protein E1A-F differentially activates matrix metalloproteinase promoters, including collagenase-1, and confers an invasive phenotype in human breast cancer cells (Kaya et al, 1996), which can be reversed by expression of antisense E1A-F (Hida et al, 1997). In addition to its effect on MMP expression, PEA3 sites influence the expression of other genes in mammary tumours. For example, a PEA3 site participates in the activation of the vimentin promoter in MDA231 whose gene product, in turn, contributes to the metastatic potential of mammary tumours (Chen et al, 1996).

Inhibition of collagenase-1 transcription by RA has been shown to be ligand dependent and to require RAR homodimers (Nicholson et al, 1990; Schule et al, 1991) and RAR/RXR heterodimers (Pan et al, 1995). In MDA231 cells, RA induces RAR-β but does not affect the expression of RAR-α and RAR-γ, which is constitutive. These findings are in agreement with others who have observed that RAR-α and RAR-γ are not regulated by RA in several human breast cancer cell lines (Roman et al, 1992), suggesting that RAR-β may be a key player in RA-mediated repression of collagenase-1 in MDA231 breast cancer cells. One possibility may be that, in the presence of ligand, the binding of RARs/RXRs to Ets-related proteins converts a potential inducer of MMP gene expression to a negative regulator, as seen with ERM in the presence of the androgen receptor (Schneikert et al, 1996).

In summary, the RA-mediated negative regulation of collagenase-1 described in this paper suggests that the mechanism(s) controlling this repression in the MDA231 breast cancer cell line differs from that observed in fibroblasts, but may resemble the steroid-hormone-mediated repression of collagenase-1 in prostate cancer cells. These mechanisms implicate PEA3 sites, rather than AP-1 sites, as major players. Thus, the AP-1 independent repression of collagenase-1 by RA implies a new repertoire of possibilities by which MMP gene expression is regulated in a cell and/or tissue-specific fashion. Our data also suggests that the therapeutic effects of retinoids may be beneficial in oestrogen-receptor-negative cell lines by inhibiting collagenase-1 expression, thereby limiting the degradation of the extracellular matrix that is associated with tumour invasion (Dickson et al, 1994; Ohishi et al, 1995).

### ACKNOWLEDGEMENTS

This study was supported by NIH, AR-26599 and the RGK Foundation (to CEB), NIH, HL-52243 (to CHL), and by fellowships from NIH: T32-CA-09658 and NRSA 1F32-AR-08437 (to UB).

### REFERENCES

Agadir A, Shealy YF, Hill DL and Zhang X (1997) Retinyl methyl ether down-regulates activator protein 1 transcriptional activation in breast cancer cells. *Cancer Res* **57**: 3444–3450

Anzano MA, Byers SW, Smith JM, Peer CW, Mullen LT, Brown CC, Roberts AB and Sporn MB (1994) Prevention of breast cancer in the rat with 9-*cis*-retinoic acid as a single agent and in combination with tamoxifen. *Cancer Res* **54**: 4614–4617

Ausuble FF, Brent R, Kingston RE, Moore DDS JG, Smith JA and Struhl K (1987) *Current Protocols in Molecular Biology*. Greene and Wiley Interscience: New York

Bae SN, Arand G, Azzam H, Pavasant P, Torri J, Frandsen TL and Thompson EW (1993) Molecular and cellular analysis of basement membrane invasion by human breast cancer cells in Matrigel-based in vitro assays. *Breast Cancer Res Treat* **24**: 241–255

Baert JL, Monte D, Musgrove EA, Albagli O, Sutherland RL and de Launoit Y (1997) Expression of the PEA3 group of ETS-related transcription factors in human breast-cancer cells. *Int J Cancer* **70**: 590–597

Benbow U and Brinckerhoff CE (1997) The AP-1 Site and MMP gene Regulation: what Is All the Fuss About? *Matrix Biol* **15**: 519–526

Benz CC, O'Hagan RC, Richter B, Scott GK, Cang CH, Xiong X, Chew K, Ljung BM, Edgerton S, Thor A and Hassell JA (1997) HER2/Neu and Ets transcription activator PEA3 are coodinately upregulated in human breast cancer. *Oncogene* **15**: 1513–1525

Birkedal-Hansen H (1995) Proteolytic remodeling of extracellular matrix. *Curr Opinions Cell Biol* **7**: 728–735

Brinckerhoff CE, Ruby PL, Austin SD, Fini ME and White HD (1987) Molecular cloning of human synovial cell collagenase and selection of a single gene from genomic DNA. *J Clin Invest* **79**: 542–546

Caelles C, Gonzalez-Sancho JM and Munoz A (1997) Nuclear hormone receptor anatgonism with AP-1 by inhibition of the JNK pathway. *Genes Dev* **11**: 3351–3364

Castaigne S, Chomienne C, Daniel MT, Ballerini P, Berger R, Fenaux P and Degos L (1990) All-*trans* retinoic acid as a differentiation therapy for acute promyelocytic leukemia. I. Clinical results (see comments). *Blood* **76**: 1704–1709

Chen JH, Vercamer C, Li Z, Paulin D, Vandenbunder B and Stehelin D (1996) PEA3 transactivates vimentin promoter in mammary epithelial and tumor cells. *Oncogene* **13**: 1667–1675

Costa A (1993) Breast cancer chemoprevention. *Eur J Cancer* **29A**: 589–592

Crawford HC and Matrisian LM (1994) Tumor and stromal expression of matrix metalloproteinases and their role in tumor progression. *Invasion Metastasis* **14**: 234–245

Crawford HC and Matrisian LM (1996) Mechanisms controlling the transcription of matrix metalloproteinase genes in normal and neoplastic cells. *Enzyme Protein* **49**: 20–37

Dickson RB, Shi YE and Johnson MD (1994) Matrix-degrading proteases in hormone-dependent breast cancer. *Breast Cancer Res Treat* **31**: 167–173

Doyle GAR, Pierce RA and Parks WC (1997) Transcriptional induction of collagenase-1 in differentiated and monocyte-like (U937) cells is regulated by AP-1 and an upstream CEBP-β site. *J Biol Chem* **272**: 11840–11849

Elder JT, Forrester WC, Thompson C, Mager D, Henthorn P, Peretz M, Papayannopoulou T and Groudine M (1990) Translocation of an erythroid-specific hypersensitive site in deletion-type hereditary persistence of fetal hemoglobin. *Mol Cell Biol* **10**: 1382–1389

Gutman A and Wasylyk B (1990) The collagenase gene promoter contains a TPA and oncogene-responsive unit encompassing the PEA3 and AP-1 binding sites. *EMBO J* **9**: 2241–2246

Heppner KJ, Matrisian LM, Jensen RA and Rodgers WH (1996) Expression of most matrix metalloproteinase family members in breast cancer represents a tumor-induced host response. *Am J Pathol* **149**: 273–282

Hida K, Shindoh M, Yasuda M, Hanzawa M, Funaoka K, Kohgo T, Amemiya K, Totsuka Y, Yoshida K and Fujinaga K (1997) Antisense E1AF transfection restrains oral cancer invasion by reducing matrix metalloproteinase activities. *Am J Pathol* **150**: 2125–2132

Higashino F, Yoshida K, Noumi T, Seiki M and Fujinaga K (1995) Ets-related protein E1A-F can activate three different matrix metalloproteinase gene promoters. *Oncogene* **10**: 1461–1463

Hong WK and Lippman SM (1995) Cancer chemoprevention. *J Natl Cancer Inst Monogr* **17**: 49–53

Jeffrey J (1986) *Regulation of Matrix Accumulation*. Mecham RP (ed.), pp. 53–98 Academic Press: New York

Kaya M, Yoshida K, Higashino F, Mitaka T, Ishii S and Fujinaga K (1996) A single ets-related transcription factor, E1AF, confers invasive phenotype on human cancer cells. *Oncogene* **12**: 221–227

Lafyatis R, Kim SJ, Angel P, Roberts AB, Sporn MB, Karin M and Wilder RL (1990) Interleukin-1 stimulates and all-*trans*-retinoic acid inhibits collagenase gene expression through its 5′ activator protein-1-binding site. *Mol Endocrinol* **4**: 973–980

Lippman SM, Heyman RA, Kurie JM, Benner SE and Hong WK (1995) Retinoids and chemoprevention: clinical and basic studies. *J Cell Biochem* (suppl.) **22**: 1–10

Lotan R (1980) Effects of Vitamin A and its analogs (retinoids) on normal and neoplastic cells. *Biochem Biophys Acta* **605**: 33–90

Lowrey CH, Bodine DM and Nienhuis AW (1992) Mechanism of DNase I hypersensitive site formation within the human globin locus control region. *Proc Natl Acad Sci USA* **89**: 1143–1147

MacDougall JR and Matrisian LM 1995) Contributions of tumor and stromal matrix metalloproteinases to tumor progression, invasion and metastasis. *Cancer Metastasis Rev* **14**: 351–362

Mangelsdorf DJ, Umesono K and Evans RM (1994) The retinoid receptors. In *The Retinoids: Biology, Chemistry and Medicine*. Sporn MB, Roberts AB and Goodman DS (eds.), pp. 319–349. Raven Press: New York

Mangelsdorf DJ, Thummel C, Beato M, Herrlich P, Schutz G, Umesono K, Blumberg B, Kastner P, Mark M and Chambon P (1995) The nuclear receptor superfamily: the second decade. *Cell* **83**: 835–839

Moon RC, Mehta RG and Detrisac CJ (1992) Retinoids as chemopreventive agents for breast cancer. *Cancer Detect Prev* **16**: 73–79

Nagase H (1996) *Zinc Metalloproteinses in Health and Disease*. NM Hopper (ed.), pp. 153–204. Tayler and Francis: London

Nicholson RC, Mader S, Nagpal S, Leid M, Rochette-Egly C and Chambon P (1990) Negative regulation of the rat stromelysin gene promoter by retinoic acid is mediated by an AP1 binding site. *EMBO J* **9**: 4443–4454

Ohishi K, Fujita N, Morinaga Y and Tsuruo T (1995) H-31 human breast cancer cells stimulate type I collagenase production in osteoblast-like cells and induce bone resorption. *Clin Exp Metastasis* **13**: 287–295

Ohuchi E, Imau K, Fujii, Y and Sato H (1997) Membrane type 1 matrix metalloproteinase digest interstitial collagenase and other extracellular matrix macromolecules. *J Biol Chem* **272**: 2446–2451

Pan L, Chamberlain SH, Auble DT and Brinckerhoff CE (1992) Differential regulation of collagenase gene expression by retinoic acid receptors-alpha, beta and gamma. *Nucleic Acids Res* **20**: 3105–3111

Pan L, Eckhoff C and Brinckerhoff CE (1995) Suppression of collagenase gene expression by all-*trans* and 9-*cis* retinoic acid is ligand dependent and requires both RARs and RXRs. *J Cell Biochem* **57**: 575–589

Pastorino U, Infante M, Maioli M, Chiesa G, Buyse M, Firket P, Rosmentz N, Clerici M, Soresi E, Valente M et al (1993) Adjuvant treatment of stage I lung cancer with high-dose vitamin A (see comments). *J Clin Oncol* **11**: 1216–1222

Roman SD, Clarke CL, Hall RE, Alexander IE and Sutherland RL (1992) Expression and regulation of retinoic acid receptors in human breast cancer cells. *Cancer Res* **52**: 2236–2242

Rutter JL, Benbow U, Coon CI and Brinckerhoff CE (1997) Cell-type specific regulation of human interstitial collagenase-1 gene expression in human fibroblasts and BC-8701 breast cancer cells. *J Cell Biochem* **66**: 11–15

Salbert G, Fanjul A, Piedrafita FJ, Lu XP, Kim SJ, Tran P and Pfahl M (1993) Retinoic acid receptors and retinoid X receptor alpha down-regulate the transforming growth factor-beta 1 promoter by antagonizing AP-1 activity. *Mol Endocrinol* **7**: 1347–1356

Sambrook J, Fritsch EF and Maniates T (1993) *Molecular Cloning: A Laboratory Manual*. Cold Spring Habor Laboratory Press: Cold Spring Harbor, NY

Schneikert J, Peterziel H, Defossez PA, Klocker H, Launoit Y and Cato AC (1996) Androgen receptor-Ets protein interaction is a novel mechanism for steroid hormone-mediated down-modulation of matrix metalloproteinase expression. *J Biol Chem* **271**: 23907–23913

Schroen DJ and Brinckerhoff CE (1996*a*) Nuclear hormone receptors inhibit matrix metalloproteinase (MMP) gene expression through diverse mechanisms. *Gene Expression* **6**: 197–207

Schroen DJ and Brinckerhoff CE (1996*b*) Inhibition of rabbit collagenase (matrix metalloproteinase-1; MMP-1) transcription by retinoid receptors: evidence for binding of RARs/RXRs to the –77 AP-1 site through interactions with c-Jun. *J Cell Physiol* **169**: 320–332

Schuchard M, Landers JP, Sandhu NP and Spelsberg TC (1993) Steroid hormone regulation of nuclear proto-oncogenes. *Endocr Rev* **14**: 659–669

Schule R, Rangarajan P, Yang N, Kliewer S, Ransone LJ, Bolado J, Verma IM and Evans RM (1991) Retinoic acid is a negative regulator of AP-1-responsive genes. *Proc Natl Acad Sci USA* **88**: 6092–6096

Seewaldt VL, Johnson BS, Parker MB, Collins SJ and Swisshelm K (1995) Expression of retinoic acid receptor beta mediates retinoic acid-induced growth arrest and apoptosis in breast cancer cells. *Cell Growth Differ* **6**: 1077–1088

Sheikh MS, Shao ZM, Li XS, Dawson M, Jetten AM, Wu S, Conley BA, Garcia M, Rochefort H and Fontana JA (1994) Retinoic-resistant estrogen receptor-negative human breast carcinoma cells transfected with retinoic acid receptor-alpha acquire sensitivity to growth inhibition by retinoids. *J Biol Chem* **269**: 21440–21447

Stamatoyannopoulos JA, Goodwin A, Joyce T and Lowrey CH (1995) NF-E2 and GATA binding motifs are required for the formation of DNase I hypersensitive site 4 of the human beta-globin locus control region. *EMBO J* **14**: 106–1016

Stetler-Stevenson WG, Liotta LA and Kleiner Jr DE (1993) Extracellular matrix 6: role of matrix metalloproteinases in tumor invasion and metastasis. *FASEB J* **7**: 1434–1441

Sugarbaker EV (1981) Patterns of metastasis in human malignancies. In *Cancer Biology Reviews*. Marchalonis JJ, Hanna MG, and Fidler IJ, (eds.), pp. 235–278. Marcel Dekker: New York

Templeton NS, Brown PD, Levy AT, Margulies IM, Liotta LA and Stetler-Stevenson WG (1990) Cloning and characterization of human tumor cell interstitial collagenase. *Cancer Res* **50**: 5431–5437

van der Burg B, Slager-Davidov R, van der Leede BM, de Laat SW and van der Saag PT (1995) Differential regulation of AP1 activity by retinoic acid in hormone-dependent and -independent breast cancer cells. *Mol Cell Endocrinol* **112**: 143–152

Vincenti MP, White LA, Schroen DJ, Benbow U and Brinckerhoff CE (1996) Regulating expression of the gene for matrix metalloproteinase-1 (collagenase): mechanisms that control enzyme activity, transcription and mRNA stability. *Crit Rev Eukaryote Gene Expression* **6**: 391–411

Wasylyk B, Hahn SL and Giovane A (1993) The Ets family of transcrition factors. *Eur J Biochem* **211**: 7–18

White LA and Brinckerhoff CE (1995) Two AP-1 elements in the collagenase (MMP-1) promoter have differential effects on transcription and bind JunD, c-Fos, and Fra-2. *Matrix Biol* **14**: 715–725

Woessner Jr JF (1991) Matrix metalloproteinases and their inhibitors in connective tissue remodeling. *FASEB J* **5**: 2145–2154

Yang-Yen HF, Zhang XK, Graupner G, Tzukermann M, Sakamoto B, Karin M and Pfahl M (1991) Anatgonism between retinoic acid receptors and AP-1: implication for tumor promotion and inflammation. *New Biol* **3**: 1206–1219

*© Cancer Research Campaign 1999*
